# Subsequent fracture risk in Norwegians and immigrants with an index forearm fracture: a cohort study

**DOI:** 10.1007/s11657-024-01419-x

**Published:** 2024-08-06

**Authors:** Sepideh Semsarian, Tone K. Omsland, Espen Heen, Ahmed Ali Madar, Frede Frihagen, Jan-Erik Gjertsen, Lene B. Solberg, Wender Figved, Jens-Meinhard Stutzer, Tove T. Borgen, Camilla Andreasen, Ann Kristin Hansen, Åshild Bjørnerem, Cecilie Dahl

**Affiliations:** 1 Department of Community Medicine and Global Health, Institute of Health and Society, University of Oslo, 0318 Oslo, Norway; 2 Institute of Clinical Medicine, University of Oslo, 0372 Oslo, Norway; 3 Department of Orthopaedic Surgery, Østfold Hospital Trust, 1714 Grålum, Norway; 4 Department of Clinical Medicine, University of Bergen, 5007 Bergen, Norway; 5 Department of Orthopaedic Surgery, Haukeland University Hospital, 5021 Bergen, Norway; 6 Division of Orthopaedic Surgery, Oslo University Hospital, 0424 Oslo, Norway; 7 Department of Orthopaedic Surgery, Vestre Viken Hospital Trust, Bærum Hospital, 1346 Gjettum, Norway; 8 Department of Orthopaedic Surgery, Møre and Romsdal Hospital Trust, Hospital of Molde, 6412 Molde, Norway; 9 Department of Rheumatology, Vestre Viken Hospital Trust, Drammen Hospital, 3004 Drammen, Norway; 10 Department of Orthopaedic Surgery, University Hospital of North Norway, 9038 Tromsø, Norway; 11 Department of Clinical Medicine, UiT The Arctic University of Norway, Post Office Box 6050, 9037 Langnes, Tromsø Norway; 12 Department of Obstetrics and Gynecology, University Hospital of North Norway, 9038 Tromsø, Norway; 13 Norwegian Research Centre for Women’s Health, Oslo University Hospital, 0424 Oslo, Norway; 14 Department of Public Health Science, Institute of Health and Society, University of Oslo, 0318 Oslo, Norway

**Keywords:** Country of birth, Ethnicity, Forearm fracture, Norway, Region of origin, Subsequent fracture risk

## Abstract

***Summary*:**

The current study investigated subsequent fracture risk following a forearm fracture in three country of birth categories: Norway, Europe and North America, and other countries. Subsequent fracture risk was modestly higher in Norwegian-born individuals compared to the two other groups. Secondary fracture prevention should be recommended regardless of country background.

**Background:**

Fracture risk is higher in patients with a previous fracture, but whether subsequent fracture risk differs by origin of birth is unknown. This study explores subsequent fracture risk in patients with an index forearm fracture according to region of birth.

**Methods:**

Nationwide data on forearm fractures in patients ≥ 18 years in 2008–2019 were obtained from the Norwegian Patient Registry and Statistics Norway. Index fractures were identified by ICD-10 code S52, whereas subsequent fractures included any ICD-10 fracture code. Data on country of birth were from Statistics Norway and included three regional categories: (1) Norway, (2) other Europe and North America and (3) other countries. Direct age standardization and Cox proportional hazard regression were used to analyse the data.

**Results:**

Among 143,476 individuals with an index forearm fracture, 35,361 sustained a subsequent fracture. Norwegian-born forearm fracture patients had the highest subsequent fracture rates (516/10,000 person-years in women and 380 in men). People born outside Europe and North America had the lowest rates (278/10,000 person-years in women and 286 in men). Compared to Norwegian-born individuals, the hazard ratios (HRs) of subsequent fracture in individuals from Europe and North American were 0.93 (95% CI 0.88–0.98) in women and 0.85 (95% CI 0.79–0.92) in men. The corresponding HRs in individuals from other countries were 0.76 (95% CI 0.70–0.84) in women and 0.82 (95% CI 0.74–0.92) in men.

**Conclusion:**

Individuals born outside Norway had a lower subsequent fracture risk than Norwegian-born individuals; however, subsequent fracture risk increased with age in all groups. Our results indicate that secondary fracture prevention should be recommended regardless of region of origin.

**Supplementary Information:**

The online version contains supplementary material available at 10.1007/s11657-024-01419-x.

## Introduction

Osteoporosis, which is the most prevalent bone disorder globally, is characterized by reduced bone mass and strength, and an increased risk of low-energy fractures [1]. Osteoporotic fractures constitute an important public health problem, especially among the elderly. Statistics show that osteoporotic fractures affect 50% of women and 20% of men in high-income countries during their lifetime [2]. The incidence rates and risks of sustaining fractures vary in different parts of the world, with a higher occurrence in high-income countries such as Northern Europe and Northern America compared to Latin America, Africa and Asia [3, 4]. Among Asian countries, Taiwan, Japan, Singapore, Kuwait, Iran and Oman are classified as high-risk nations for fractures [5, 6]. The global demographic landscape is undergoing significant changes, particularly in Asia, where the ageing population has led to a notable increase in the incidence of fractures. In Europe, a discernible north–south disparity in fracture risk exists, with northern and Scandinavian countries reporting higher prevalence and incidence rates compared with countries in mainland Europe [4, 7]. Previous studies have shown that the incidence rates of forearm and hip fractures in Norwegian citizens are among the highest worldwide [3, 5, 8].

Fractures, particularly subsequent ones, often lead to decreased mobility, functional challenges, difficulties in work and social activities, and increased mortality risk [9]. The most common osteoporotic fractures occur in the distal forearm, hip and spine [10]. Fractures are also common among children, adolescents and young adults without osteoporosis [11]. Fractures in young adults occur more often in men, frequently as a result of high-energy trauma like car accidents and sports activities [12].

Suffering a forearm fracture almost doubles the risk of a subsequent osteoporotic fracture [13]. The risk of a subsequent hip fracture, which is the most serious type of osteoporotic fracture, varies from 2 to 20% in different studies [14, 15]. A study conducted in Sydney, Australia, examined the risk of subsequent fracture after a low-energy fracture with the International Classification of Diseases, 10th Revision (ICD-10) codes S22–S82: fracture of rib(s), lumbar spine, shoulder, and upper arm, forearm, wrist, femur and hip, and lower leg [16]. They found a cumulative incidence of 7.1% at 1 year and 13.7% at 5 years after the initial fracture for women, and 6.2% at 1 year and 11.3% at 5 years for men [16].

There is a wide range of risk factors contributing to fractures and subsequent fractures (e.g. age, sex, prior fracture, low bone mineral density (BMD), smoking, nutritional status and family history of fractures [11, 12]). In general, the risk of sustaining a subsequent fracture increases with age [8, 24]. For example, a previous study from Tromsø, Norway, which included 3108 individuals with an initial fracture after the age of 49 found that the risk of sustaining a subsequent fracture of any type in women increased from 9 to 30% between the age groups of 50–59 and 80 + [24]. For men in the same age range, the risk increased from 10 to 26%. Notably, 26% of women and 18% of men over 80 years old sustained subsequent fractures, regardless of their increased risk of mortality [24]. Studies have shown that childhood fractures are associated with low bone mineral density (i.e. possibly due to reduced peak bone mass), and the risk of future skeletal fragility and future fractures in adulthood [17]. Some studies indicate that the country of origin or ethnicity may represent a risk factor for fracture [18, 19], but the reasons are not fully understood. Bone mineral density, bone microarchitecture, bone strength and factors related to the risk of falling vary in populations in different geographical areas, probably due to both genetic and environmental causes, but none of these factors alone can explain the differences in fracture risk [20, 21].

To our knowledge, subsequent fracture risk according to region of origin has not previously been studied. The aim of this study was therefore to estimate the association between region of origin and the risk of any recurrent fracture in patients with an index forearm fracture, which is the most common fracture type in Norway.

## Materials and methods

### Study population and data sources

This cohort study included all Norwegian residents aged 18 and older seeking fracture treatment between 2008 and 2019. Within this period, all individuals who sustained an index forearm fracture (any ICD-10 S52 coded fracture, including both high-energy and low-energy fractures) treated at the hospitals and large emergency rooms across Norway were included in the dataset and observed for any subsequent fractures. Data on fractures was obtained from the Norwegian Patient Registry (NPR), while data on migration, marital status and country of birth was obtained from Statistics Norway.

### Definition of the outcome: subsequent fractures

All types of fractures were defined through standardized ICD-10 codes for diagnosis in the Norwegian health care system: fracture of rib(s), sternum and thoracic spine (S22), lumbar spine and pelvis (S32), shoulder and upper arm (S42), forearm (S52), wrist and hand (S62), hip and femur (S72), lower leg, including ankle (S82), and foot except ankle (S92), including all subcategories. We excluded registrations of follow-up visits, except for first-time registrations with a code for follow-up examination, as some patients with fractures receive initial treatment in primary care (not reporting to the (NPR)) before being referred to the hospital, and consequently, incident fractures are sometimes coded as a follow-up visit [22]. A wash-out period of 6 months (within each fracture type) was applied to handle multiple registrations regarding the same fracture. Records with surgical coding for reoperation were also omitted. Our algorithm for identifying forearm fractures was recently validated, and it has a sensitivity of approximately 90% and a positive predictive value of 90% [23].

### Observation time

The maximum observation time in the study was 12 years, from January 1, 2008, through December 31, 2019. All individuals experiencing an index forearm fracture (any S52 fracture) were included in the study and followed for any type of subsequent fracture. Person-time in the analyses was calculated as the time from index forearm fracture to the subsequent fracture or censoring (emigration, death or end of study).

### Main exposures categories

Individuals were categorized into three main groups of countries of birth (pre-defined categories from Statistics Norway): (1) Norway, (2) other European countries and North America and (3) other countries, including Central and Southeast Asia, Africa, the Middle East, and Central and South America. We excluded 990 individuals with missing information about their country of origin (0.69% of the total population). The remaining population included in the study was 143,476 individuals with an index forearm fracture (S52). The individuals were stratified according to age: 18–44 years, 45–59 years and 60 years and older, to study the risk in different age groups. In sensitivity analyses, the individuals were also stratified according to marital status (not married/married) and education level (< 12 years/ ≥ 12 years).

### Statistical analyses

Descriptive and survival analyses were performed in Stata 16. Age-standardized incidence rates (IRs) were calculated as the number of fractures divided by the number of years at risk after the first fracture using a direct standardization method (with the mean distribution of age during follow-up between 2008 and 2019 in the Norwegian-born population with an index forearm fracture as standard). The results were reported as the number of fractures per 10,000 person-years. Cox proportional hazard models were used to calculate the risk of subsequent fracture as a function of country of birth, divided into the three main geographical regions of origin groups, adjusted for age differences and stratified by sex. log minus log curves were evaluated regarding the assumption of proportional hazards, and the assumptions were considered fulfilled. Age-adjusted hazard ratios (HRs) and corresponding 95% confidence intervals (CIs) were obtained from the models. Two-sided *p* values < 0.05 were considered significant.

### Ethics

The current study and the linkage of data from the Norwegian Patient Registry to Statistics Norway were approved by the Regional Committee for Medical and Health Research Ethics (REC), with application number 2015/334 and reference number 26953, and the Directorate of Health, with reference number 17/25552–37. The University of Oslo performed a Data Protection Impact Assessment (DPIA) in accordance with the General Data Protection Regulation.

## Results

Among the 143,476 individuals with an index forearm fracture included in the study, 42,923 were men and 100,553 were women. Of the total, 127,431 individuals were born in Norway, 10,537 in other European countries or North America and 5508 in other countries, with a mean age at first forearm fracture of 59.0 years, 48.6 years and 44.3 years, respectively (Table 1). Among the included individuals, 35,361 (24.6%) sustained a subsequent fracture of any type. The number of person-years for the whole cohort of forearm fracture individuals was 767,531, with a total IR for subsequent fracture of 461 (95% CI 456–466) per 10,000 person-years.

**Table 1:** Number of individuals aged 18 + years in 2008–2019 with an index forearm fracture and any type of subsequent fracture categorized by country of birth (three main groups), and mean age with 95% confidence interval (CI) at the index fracture

Country of birth	Index forearm fracture (*N*)	Mean age	95% CI	Subsequent fractures (*N*)
Norway	127,431	59.0	58.9–59.1	32,664
Europe and North America	10,537	48.6	48.3–48.9	1899
Other countries	5508	44.3	43.9–44.7	798
Total	143,476	57.7	57.6–57.8	35,361

### Subsequent fractures according to region of origin

Norwegian-born women had the highest IR of a subsequent fracture (516 per 10,000 person-years), whereas IRs among women born in other European countries or North America and born in other countries were 406 and 278 per 10,000 person-years, respectively (Table 2). Norwegian-born men had the highest subsequent fracture IR (380 per 10,000 person-years), while the IRs for men born in other European countries or North America and other countries were 303 and 286 per 10,000 person-years, respectively (Table 2).

**Table 2:** Number of participants aged 18 + years in 2008–2019 with an index forearm fracture, number of subsequent fractures of any type, age-standardized incidence rates (IRs) per 10,000 person-years and age-adjusted hazard ratio (HR) of subsequent fracture with 95% CI for the different countries of birth (three main groups) stratified on sex

	No. of index forearm fractures	No. of subsequent fractures	IR	95% CI	HR	95% CI
Women	100,553	26,654				
Norway	91,409	24,979	516	509–522	1	Reference
Europe and North America	6007	1215	406	384–429	0.93	0.88–0.98
Other countries	3137	460	278	254–304	0.76	0.70–0.84
Men	42,923	8707				
Norway	36,022	7685	380	372–389	1	Reference
Europe and North America	4530	684	303	281–326	0.85	0.79–0.92
Other countries	2371	338	286	257–318	0.82	0.74–0.92

Compared to Norwegian-born women, the age-adjusted HR for any type of subsequent fracture was 0.93 (95% CI 0.88–0.98) in women from other European and North American countries (Table 2). The HR of any subsequent fracture among women born in other countries was 0.76 (95% CI 0.70–0.84) compared to that among Norwegian-born women. The HR of subsequent fractures was 0.85 (95% CI 0.79–0.92) in men born in European and North American countries compared to that in Norwegian-born men. The HR of risk of any subsequent fracture among men born in other countries was 0.82 (95% CI 0.74–0.92) compared to that among Norwegian-born men (Table 2).

### Subsequent fractures according to age

The risk of subsequent fractures increased with age, irrespective of the region of birth. Incidence rates increased from 280 per 10,000 person-years among Norwegian-born individuals aged 18–44 years to 616 per 10,000 person-years among Norwegian-born individuals aged over 60 years (Supplementary Table 1). Norwegian-born women aged over 60 years had the highest IR (640 per 10,000 person-years), while the lowest IR was among women aged 18–44 from other countries (226 per 10,000 person-years) (Table 3).

**Table 3:** Number of participants aged 18 + years in 2008–2019 stratified by sex, age and the three regions of origin, .Number of subsequent fractures of any type, age-standardized IRs per 10,000 person-years and age-adjusted HR of subsequent fracture with 95% CI

	No. of index forearm fracture	No. of subsequent fracture	IR	95% CI	HR	95% CI
Women						
Ages 18–44	16,882	2483				
Norway	13,645	2074	246	236–257	1	Reference
Europe and North America	1872	242	255	225–289	1	0.88–1.14
Other countries	1365	167	226	194–263	0.88	0.76–1.04
Ages 45–59	25,930	6386				
Norway	22,865	5850	438	427–449	1	Reference
Europe and North America	1876	342	358	322–398	0.82	0.74–0.92
Other countries	1189	194	311	270–358	0.72	0.62–0.83
Age + 60	57,741	17,785				
Norway	54,899	17,055	640	631–650	1	Reference
Europe and North America	2259	631	580	536–627	0.95	0.88–1.03
Other countries	583	99	339	279–413	0.63	0.52–0.77
Men						
Ages 18–44	17,665	3183				
Norway	13,325	2561	314	302–326	1	Reference
Europe and North America	2790	402	286	259–315	0.86	0.78–0.96
Other countries	1550	220	284	249–325	0.86	0.75–0.99
Ages 45–59	11,782	2302				
Norway	9930	2016	338	323–353	1	Reference
Europe and North America	1239	194	318	277–367	0.89	0.77–1.04
Other countries	613	92	292	238–358	0.83	0.67–1.02
Age + 60	13,476	3222				
Norway	12,767	3108	510	492–528	1	Reference
Europe and North America	501	88	357	289–440	0.77	0.62–0.95
Other countries	208	26	281	191–413	0.63	0.43–0.93

**Fig. 1:**
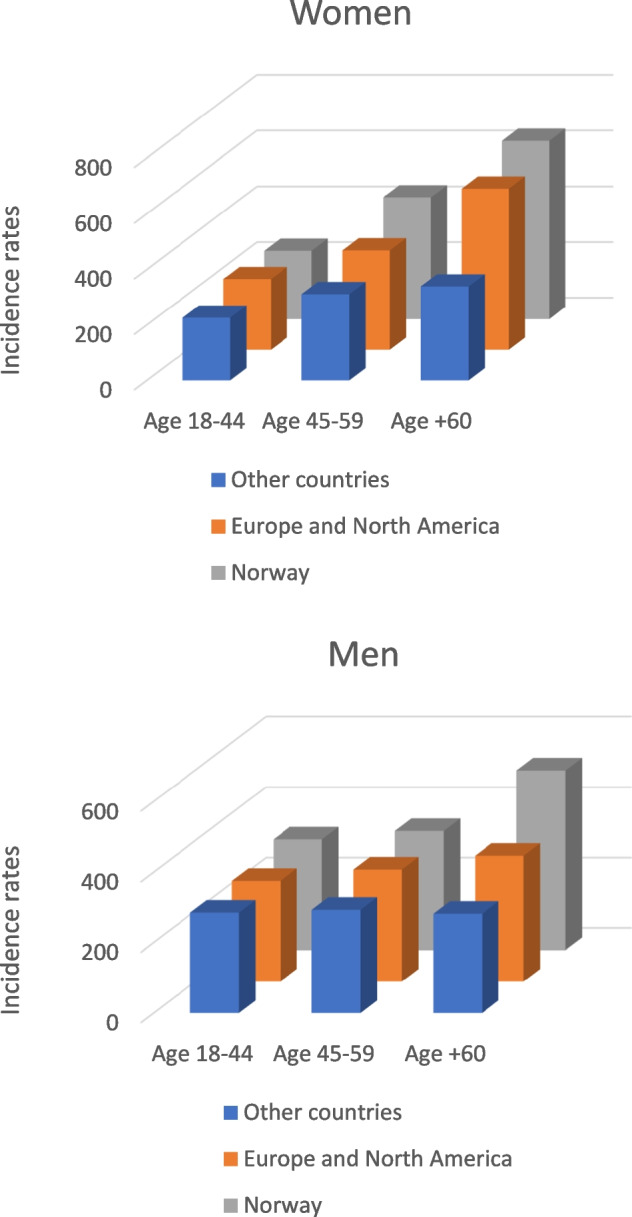
Age-standardized incidence rates (IRs) of subsequent fractures per 10,000 person-years for individuals by age and region of origin, stratified on sex

Incidence rates of subsequent fractures among Norwegian-born men and men from other European countries and North America also increased with age, whereas incidence rates among men from other countries showed less variation with age. Among all men, Norwegian-born men aged over 60 years had the highest IR (510 per 10,000 person-years) (Table 3 and Fig. 1). Subsequent fracture risk in immigrants from other countries was significantly lower in all age groups compared to Norwegian-born individuals (Table 3).

### Types of subsequent fractures

**Fig. 2:**
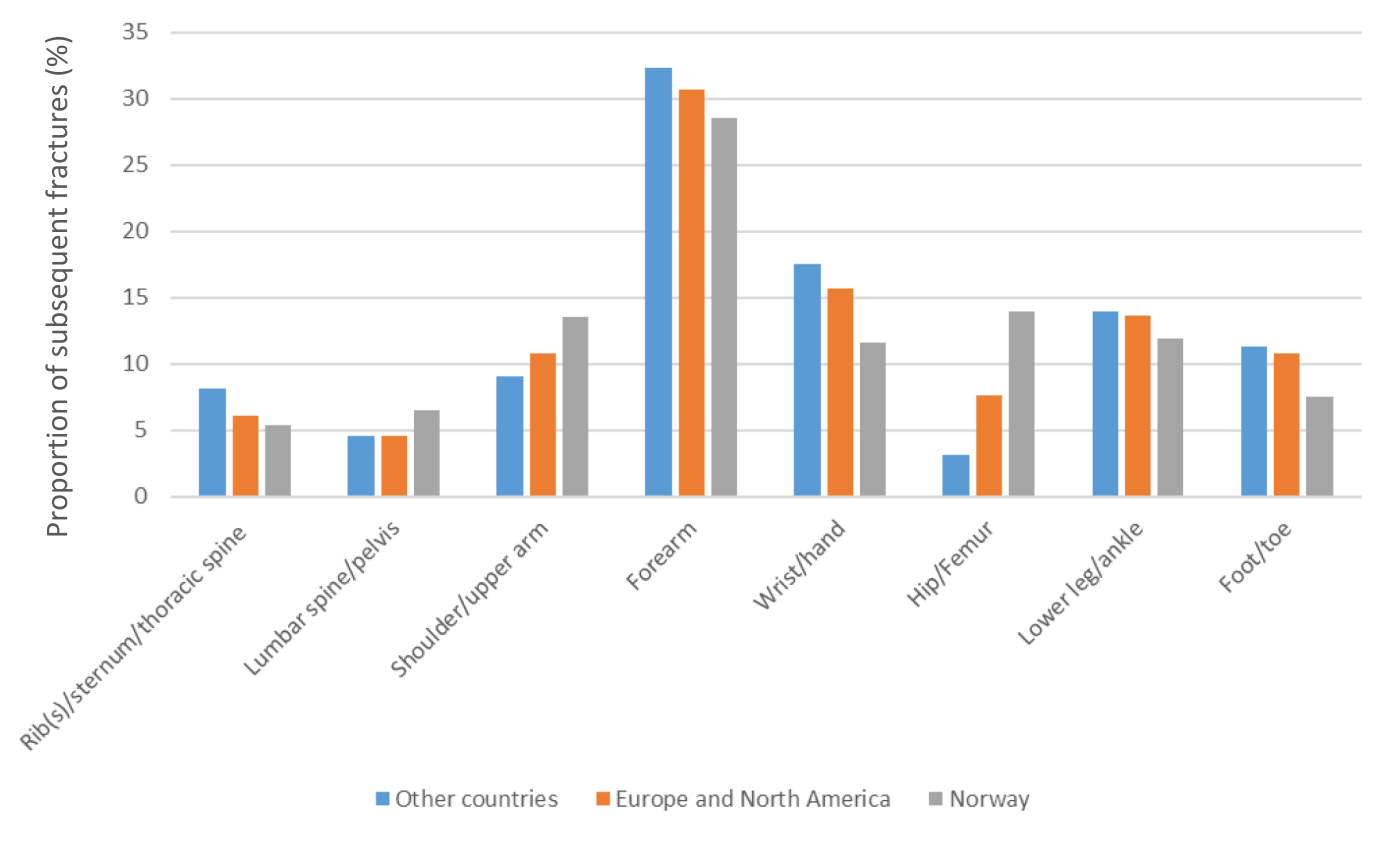
Distribution of subsequent fractures in patients with an index forearm fracture in 2008–2019 by region of origin

**Fig. 3:**
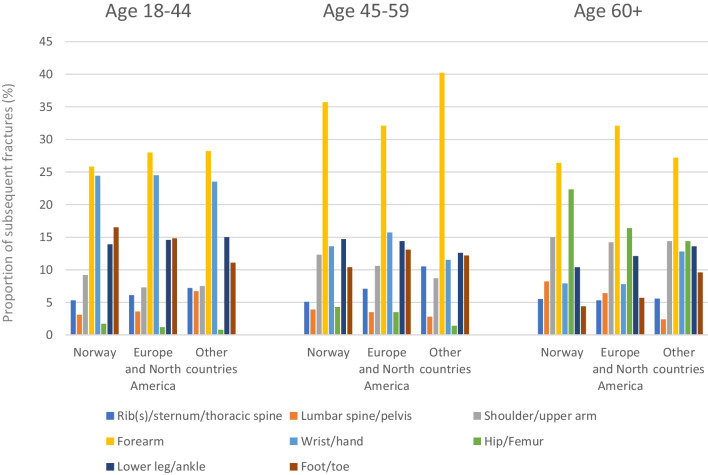
Distribution of subsequent fractures in patients with an index forearm fracture in 2008–2019 divided into region of origin and by age

A forearm fracture was the most common type of subsequent fracture in all ethnic groups (*n* = 10,165) and accounted for 28.75% of subsequent fractures, while fractures of the rib(s), sternum, spine and pelvis were the least common subsequent fractures reported to the NPR (Fig. 2). Compared to Norwegian patients, hip and femoral fractures were less common subsequent fracture types among people from other countries, while fractures of the shoulder and upper arm were more common (Fig. 2). In all individuals aged over 60 years, the second most common fracture (after a forearm fracture) was a femur/hip fracture while among individuals aged 18–44 and 45–59, a fracture of the wrist/hand and a fracture of the lower leg were more common than a hip fracture (Fig. 3). See Supplementary Fig. 1 for a depiction of the major types of low-energy fractures.

### Sensitivity analysis of subsequent fracture risk according to marital status and education level

Adjusting for marital status and education level did not change the overall pattern of the associations in Table 2. No interaction was found between marital status and region of origin. However, there was a significant interaction between education level and region of origin in women; i.e. the HR of women with low (< 12 years) education and backgrounds from countries outside Europe and North America was 0.67 (0.58, 0.78), while those with high education (≥ 12 years) had a HR of 0.84 (0.74, 0.86). The associations were also stronger in men with low education, but no significant interaction was found.

## Discussion

In this cohort study, we included almost 150,000 individuals with an index forearm fracture between 2008 and 2019 and found a higher risk of any type of subsequent fracture in Norwegian-born individuals compared to individuals born outside of Norway. The lowest risk of subsequent fracture was observed in patients in the ‘other countries’, i.e. those born outside Europe and North America. Incidence rates of subsequent fractures increased with age, regardless of country background. Although individuals from other European and North American countries had lower incidence rates and subsequent fracture risks after a forearm fracture compared to those born in Norway within the same age groups, their rates and risks remained high across all age brackets. Among all regions of origin groups, a forearm fracture was the prevailing type of subsequent fracture. Those born outside Norway also suffered forearm fractures at a younger age (44–49 years) compared to Norwegian-born patients (59 years).

A previous Norwegian study found an overall incidence of forearm fractures of 398 per 100,000 person-years [24]. If we assume that forearm fractures constitute 20% of all fractures, we can estimate the overall fracture incidence in Norway to be 199 per 10,000 person-years. Consequently, the observed subsequent fracture incidence of 461 per 10,000 person-years in the current study is 2.3 times higher than the overall fracture estimate. This shows a markedly elevated subsequent fracture risk in individuals with an index forearm fracture, contrasting sharply with the general population that has not experienced previous fractures.

Other studies has also reported that the risk of subsequent fracture is high among individuals with an initial fracture of any type [15, 25, 26]; for instance, a Norwegian study showed that women and men with previous hip fractures have a 2.5-times and 5-times higher risk of experiencing a new hip fracture, respectively [27].

Several surveys have studied the worldwide risk of fracture, especially among the aged populations [15, 26]. According to previous studies, Europe, particularly the Scandinavian countries, and North America have been considered high-risk regions for fractures [5, 28]. In contrast, countries in Latin America and Central and Southeast Asia have been considered low-risk areas [5, 28, 29]. Some studies have demonstrated statistically different incidence rates of fracture in populations with different ethnicities. It has been reported that European and North American citizens have higher incidence rates compared to individuals from Africa, Latin America and Central and Southeast Asia [28, 30]. Furthermore, a Swedish study found that the incidence rate of hip fracture among Swedish-born citizens was approximately doubled compared to the corresponding rate among immigrants [29]. It also reported that the incidence increased over time among immigrants but remained significantly lower than that in the native population [29]. The current study found a similar pattern in subsequent fracture risk among populations with different country backgrounds.

On the other hand, a recent meta-analysis focusing on subsequent fracture risk reported that the risk remained consistent across individuals from different countries of birth [31]. Nevertheless, the studies incorporating race and ethnicity had fewer person-years of follow-up, affecting the power to detect differences. Our findings highlight distinct subsequent fracture risks among individuals of diverse regions of origin. We found a higher risk of subsequent fracture of any type in Norwegian-born individuals compared to individuals born outside of Norway. Utilizing comprehensive register data from an entire country over several years, as done in the current study, provides an exceptional opportunity to explore variations in subsequent fracture risk.

The causes of the different risks of subsequent fractures are unclear. Some previous studies have suggested that countries with higher socio-economic growth have higher fracture rates, which can have a correlation with lifestyles such as sedentary lifestyles, smoking, nutrition status and alcohol consumption [3, 32].

Migration also has an impact on health. However, the effect of migration varies among different immigrant groups [33]. Over time, the risk of disease has been found to equalize with the population that they immigrate to. In Sweden, a similar risk of first osteoporotic fracture was found among second-generation immigrants and Swedish natives, probably due to environmental factors [34]. In addition, it is likely that at least some of the regional differences can be explained by differences in the proportion of cases that are diagnosed and properly recorded.

Another possible explanation for the lower risk of fractures in immigrants living in Norway compared to Norwegian-born individuals is the healthy migrant effect. This theory claims that the healthiest people in a population are most likely to migrate, initially resulting in a superior overall health condition in immigrants compared to the population of origin and the host population [28, 35]. It has been found that, overall, immigrants in Norway have an 11% survival advantage. However, some immigrant subgroups, such as refugees, might have higher mortality rates than the general Norwegian population [36].

Other factors contributing to differences in the risk of subsequent fractures in different ethnicities can be genetics and biological variations in the skeleton. Studies reported differences in the macrostructure and microstructure of bones in people from different ethnicities; Chinese and Africans have a more robust bone architecture. Chinese women have a lower fracture risk of hip and distal forearm than Caucasians, partly due to thicker cortices and thicker trabeculae [37]. Caucasians also have a lower BMD than Africans, Hispanics and Latin Americans, and the heritability of BMD is estimated between 50 and 85% [21, 37].

Population demographics are contributing factors to fracture risk; the proportion of older adults in the population is increasing in European and North American countries, leading to a higher rate of fractures in these areas. In addition, latitude and environmental factors can play a role [4]. Variation in the early environment influences peak bone mass, which is considered an important risk factor for childhood bone fractures, osteoporosis and fracture risk in later life [19, 38]. Another possibly important factor is time spent in Norway. In this study, we lacked detailed information about the duration of residency.

### Strengths and limitations

The large size of the study sample (almost 150,000 index forearm fractures) and the quality of the data are strengths of the study. The data used in the study were from patient registries with relatively reliable diagnosis codes and were further linked to other data sources by a person-identifiable number. Almost all Norwegian residents who sustained a forearm fracture aged 18 + years in 2008–2019 were included and followed for up to 12 years.

Several previous studies have focused on hip fractures since these fractures are easier to study in register-based data as surgical hospital admissions, at least in high-income countries [39]. Forearm fractures, on the other hand, can be treated in both primary and specialist care and, depending on severity, can be treated either conservatively, often in emergency units, or surgically in hospitals. The study included only individuals seeking hospitalization for the diagnosis and treatment of a fracture. We might have missed those treated only in primary care (about 5–7% of all forearm fracture patients) [22]. However, according to Statistics Norway, immigrants are more likely to live in urban areas (where fractures are reported to NPR) than in rural areas. In rural areas, although rare, individuals are more likely to be exclusively treated in primary care without a referral to a hospital; consequently, the fractures might not be captured by the registry. Thus, there was probably a relatively greater probability of missing Norwegian-born than immigrant individuals in this study [22].

We might also have missed individuals with fractures sustained abroad. However, these fractures would have been captured if they had been followed up in Norway, as we included records with follow-up codes that occurred only once in the dataset. Still, we might have missed more fractures during travel among immigrants because they are more likely to travel and stay abroad for a longer time than the Norwegian-born population. These two biases work in opposite directions on the HR and may, therefore, have limited effect on the findings of lower risk in the immigrant population. Nevertheless, the proportion of fractures missed due to travel is unknown, and we cannot rule out the possibility that some of the difference is explained by this.

Additionally, registry data has its drawbacks, as some essential information may be lost due to misclassification or changes in coding methods [40, 41]. Still, the current registry-based data was found to have high validity when using standardized algorithms for quality assurance [23].

The Norwegian-born individuals significantly outnumbered individuals from other ethnic backgrounds, which led to uncertainty in incidence rates and hazard ratios. To address this problem, the populations from other countries were categorized into one group, but still, the total numbers were relatively low and confidence intervals were wide. Moreover, the different age distributions in the Norwegian versus immigrant populations also complicated the comparisons of fracture risk, and therefore, we performed age-specific analyses in 15-year age groups.

In conclusion, we found that the risk of subsequent fracture varied by region of origin. In both men and women, there was a higher risk of subsequent fractures among Norwegian-born individuals compared to individuals born in countries outside of Norway. However, individuals born in countries outside of Norway had their first forearm fracture at a younger age. The risk of subsequent fracture increased with age in all groups, and there was a high rate of subsequent fractures also in the immigrant populations, which warrants a focus on the prevention of subsequent fractures in all ethnicities regardless of the country of birth. A forearm fracture doubles the risk of a subsequent fracture, and the younger age at fracture in immigrant groups presents an unexplored opportunity for the early prevention of future fractures. Norwegian-born individuals have among the highest risks of fracture in the world. Future studies should further focus on subsequent fracture risk in immigrants according to length of stay in Norway to elucidate whether a different early environment could be the reason why immigrant populations have a lower risk of subsequent fractures compared to Norwegian-born individuals.

## Supplementary Information

Below is the link to the electronic supplementary material.

Supplementary file1 (DOCX 38.6 KB)

Supplementary file2 (DOCX 15.5 KB)

## Data Availability

Due to the protection of privacy under General Data Protection Regulation and Norwegian law, the individual-level data can only be made available after approval by the Regional Committee for Medical and Health Research Ethics.

## Declarations

### Disclaimer

Data from the Norwegian Patient Registry has been used in this publication. The interpretation and reporting of these data are the sole responsibility of the authors, and no endorsement by the Norwegian Patient Registry is intended or should be inferred.
