# Supplementary material for: Subsequent fracture risk in Norwegians and immigrants with an index forearm fracture: a cohort study
Source: Arch Osteoporos. 2024 Aug 6;19(1):72. doi: 10.1007/s11657-024-01419-x (PMC11303429; doi:10.1007/s11657-024-01419-x)
Supplement: Supplementary file 1 — Supplementary file1 (DOCX 38.6 KB) [file 11657_2024_1419_MOESM1_ESM.docx]

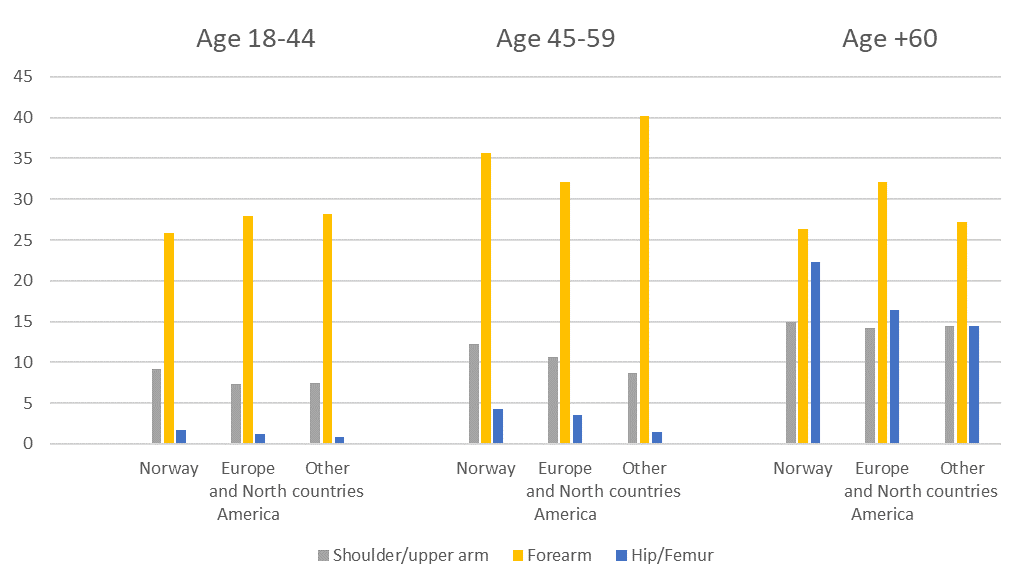


Proportion of subsequent fractures (%)

**Supplementary** **Fig 1** Distribution of major subsequent fractures in patients with an index forearm fracture 2008–2019 divided into three regions of origin and by age
