# Supplementary material for: Subsequent fracture risk in Norwegians and immigrants with an index forearm fracture: a cohort study
Source: Arch Osteoporos. 2024 Aug 6;19(1):72. doi: 10.1007/s11657-024-01419-x (PMC11303429; doi:10.1007/s11657-024-01419-x)
Supplement: Supplementary file 2 — Supplementary file2 (DOCX 15.5 KB) [file 11657_2024_1419_MOESM2_ESM.docx]

**Supplementary** **Table 1** Number of participants aged 18+ years in 2008–2019 divided into three age groups (*N*). Number of subsequent fractures, age-standardized incidence rates (IR) per 10,000 person-years, and age-adjusted hazard ratio (HR) of subsequent fracture with 95% confidence interval (CI) for the different regions of origin

|  | *N* | No. subsequent fracture | IR | 95% CI | HR | 95% CI |
| --- | --- | --- | --- | --- | --- | --- |
| Age 18-44 | **35,547** | **5,666** |  |  |  |  |
| Norway | 26,970 | 4,635 | 280 | 271-287 | 1 | (Reference) |
| Europe and North America | 4,662 | 644 | 273 | 253-295 | 0.91 | 0.84-0.99 |
| Other countries | 2,915 | 387 | 256 | 232-283 | 0.88 | 0.79-0.97 |
| Age 45-59 | **37,712** | **8,688** |  |  |  |  |
| Norway | 32,795 | 7,866 | 407 | 398-416 | 1 | (Reference) |
| Europe and North America | 3,115 | 536 | 343 | 315-373 | 0.85 | 0.78-0.93 |
| Other countries | 1,802 | 286 | 304 | 271-342 | 0.75 | 0.69-0.85 |
| Age 60+ | **71,217** | **21,007** |  |  |  |  |
| Norway | 67,666 | 20,163 | 616 | 608-625 | 1 | (Reference) |
| Europe and North America | 2,760 | 719 | 539 | 501-580 | 0.92 | 0.85-0.99 |
| Other countries | 791 | 125 | 325 | 273-388 | 0.63 | 0.53-0.75 |
